# COVID-19 Vaccination: crucial roles and opportunities for the mental health professionals

**DOI:** 10.1017/gmh.2021.25

**Published:** 2021-06-25

**Authors:** Debanjan Banerjee, Sanchari Mukhopadhyay, Mariam Sahana Asmeen, Afzal Javed

**Affiliations:** 1 Department of Psychiatry, National Institute of Mental Health and Neurosciences (NIMHANS), Bengaluru, India; 2 Department of Integrative Medicine, National Institute of Mental Health and Neurosciences (NIMHANS), Bengaluru, India; 3 Department of Psychology, University of Dhaka, Dhaka, Bangladesh; 4 Chairman Pakistan Psychiatric Research Centre, Fountain House Lahore, Pakistan & President, World Psychiatric Association (WPA), Geneva, Switzerland

**Keywords:** COVID-19 pandemic, mental health professionals, psychiatrists, public mental health, severe mental illness, vaccination

## Abstract

Besides addressing the increased prevalence of psychiatric disorders, social challenges, and building community resilience during the crisis, mental health professionals (MHPs) are in a unique position to assist the vaccination drive against coronavirus disease-2019 (COVID-19) in various nations. Vaccination programs are adversely affected by misinformation, fake news and vaccine hesitancy fuelled by social media. MHPs can enable this vital public health strategy by prioritizing vaccination for individuals with severe mental illness (SMI) and substance use disorders, promote awareness and public education, debunk misinformation and integrate psychosocial care into the vaccination drives. In order to target the health inequity and discrimination faced by people with SMI coupled with their additional risks, the authors urge the global mental health fraternity to tailor these crucial roles with respect to COVID-19 vaccination based on the regional needs and contexts.

## Premise: Psychosocial burden of COVID-19

It is more than a year of the unprecedented coronavirus disease-2019 (COVID-19) crisis. When the World Health Organization (WHO) declared COVID-19 as a public health emergency of international concern on 30th January 2020, and subsequently as a pandemic on 11th March 2020, little did we know that the causative virus severe acute respiratory syndrome coronavirus-2 (SARS-CoV-2) will bring the world down on its knees. With 140 million people affected worldwide and more than 3 million lives lost till date (as of 17th April 2021), the outbreak has affected lives and living in more ways than one (Worldometer, n.d.). The consequent lockdown has affected economies, travel and relationships, measures of social distancing, and face masks have affected ‘social touch’ and restricted emotional expressions, and finally the fear, stigma, and uncertainty due to the pandemic itself have led to a myriad of adverse psychosocial consequences (Torales *et al*., 2020). Tandon in his editorial in 2020 aptly focused on the mental health burden of the pandemic, highlighted the role of mental health professionals (MHPs), and called for ‘preserving humanity, maintaining sanity, and promoting health’ amidst the crisis (Tandon, 2020).

Historically, infectious disease outbreaks always had intersections with psychological health. Besides increasing the prevalence of psychiatric disorders such as depression, anxiety, psychosis, adjustment disorders, post-traumatic stress, and insomnia, coronavirus disease-2019 (COVID-19) and resultant situations have affected the symptom severity and treatment of people living with mental illness (Pfefferbaum and North, 2020; The Lancet Infectious Diseases, 2020; Vindegaard and Benros, 2020). Furthermore, the psychological morbidity has increased in the form of social stigma, discrimination, domestic violence, impaired psychosexual health, panic, isolation, loneliness, problems in interpersonal relationships, and bereavement (Douglas *et al*., 2020; Vindegaard and Benros, 2020). The frontline workers, elderly, and socio-economically impoverished are the vulnerable groups more prone to these effects. Also, the severe acute respiratory syndrome-coronavirus-2 (SARS-CoV-2) itself has shown to cause short-term and long-term neuropsychiatric complications, with delirium, confusional states, seizures, encephalitis, cognitive impairment, and strokes being the common ones (Banerjee and Viswanath, 2020). In the largest study so far, a 6-month retrospective cohort study using electronic health records, Taquet *et al*., reported neuropsychiatric outcomes in 2,36379 COVID-19 survivors. Nearly one-third of them had at least one such disorder, among which 12.8% received such a diagnosis for the first time in life. Severe infections and ITU admissions were related to higher incidence of neuropsychiatric complications. Ischemic strokes, parkinsonism, dementia, anxiety, and psychotic disorders were the common ones reported (Taquet *et al*., 2021a). This clearly provides an indication of the psychiatric comorbidity that has been there over the last year and warns about its likely increase as the second wave of infections emerges globally. Here comes the vital role of MHPs.

## Crucial role of the MHP

The crucial role of psychiatrists and other MHPs (clinical psychologists, psychiatric social workers, psychiatric nurses) is paramount during the ongoing crisis. Their role is not limited to addressing the increasing burden of psychiatric disorders, but also encompasses public mental health education and promotion, integration of mental healthcare at all levels, facilitation of problem-solving and crisis management, empowerment of those at high-risk, and encouragement of self-care and coping among the medical fraternity (Banerjee, 2020). MHPs also foster community resilience-building, distinguish and manage healthy *v.* unhealthy stress responses during pandemic crisis, understand human behavior and panic, deal with medical misinformation, address unhealthy lifestyle patterns and use of technology, and importantly, carry out their roles as physicians as well (Onyemaechi, 2020). Considering the present circumstances, MHPs can be entrusted with a vital yet challenging role, i.e. their involvement and responsibilities related to the COVID-19 vaccination. The professional capacity to study and modify problematic human thinking and behaviors place MHPs in a unique role to enable global vaccination drives, which are long-term processes influenced by complex human attitudes, cognitions, and socio-political dynamics.

## COVID-19 vaccines and the MHPs

The COVID-19 vaccine is intended to provide acquired immunity against SARS-CoV-2. The viral genetic sequence data were shared by GISAID on 10th January 2020 and since then there has been growing work on the development of vaccines (Thanh Le *et al*., 2020). Till date, several COVID-19 vaccines have demonstrated up to 95% efficacy in preventing symptomatic COVID-19 infections. Like any other vaccination, the goal is to prevent severe and life-threatening infections, hospitalization, and mortality. Thirteen vaccines have been authorized by the national regulatory authorities for public use in various countries as of April, 2021 (Kim *et al*., 2021, p. 19). Data from Vaccine Centre, London School of Hygiene and Tropical Medicine, 2021 show that there are 308 vaccine candidates in various stages of development with clinical research focusing on higher efficacy and coverage of mutant strains. As we speak, many nations have implemented national vaccination programs, focusing on the vulnerable populations such as the frontline workers, elderly, and immunocompromised groups. Officially published reports from global health agencies mention that as of 16th April 2021, 878.16 million COVID-19 vaccine doses have been administered worldwide (COVID-19 Vaccine Tracker, 2021). There have been active scientific discourse and debates about the extent of efficacy of vaccines, the interval between two doses, and the comparative efficacy between various types of vaccines. A detailed discussion of these aspects is not intended for this article. Notwithstanding these factors, general recommendations from global health agencies and popular research recommend vaccinations against COVID-19 (Kim *et al*., 2021, p. 19). However, national vaccination drives, and coordination and awareness about the need for vaccines are as challenging aspects in public health as the discovery and manufacturing of the vaccines themselves.

It has been well-researched how vaccination drives and coverage are prone to social stereotypes and public attitudes, which, in turn, are mediated by misinformation and disinformation (Burki, 2019). Since the advent of COVID-19, it has been termed as an ‘infodemic’ (information epidemic), with increasing rumor-mongering, fake news, and conspiracy theories about its origin, spread, necessary precautions, and management. This has been further fueled by social media and increased ‘digital screen time’ during lockdown (Banerjee and Meena, 2021). The WHO mentioned that, *‘the coronavirus disease is the first pandemic in history in which technology and social media are being used on a massive scale to keep people safe, informed, productive and connected.’* (Charpentrat, 2020). It becomes particularly critical when misinformation and personal opinions tend to go against evidence-based medicine. Vaccinations against pneumococcal infection, poliomyelitis, influenza and tuberculosis have all been challenged by medical misinformation (Burki, 2019). Vaccine hesitancy and belief that vaccines are unsafe are significantly affected by fake news, which is propagated by digital media. In a recent large cross-country study using regression framework, Wilson and Wiysonge reported that a 2-percentage point drop in mean vaccination annual coverage was caused by a 1-point shift in a 5-point misinformation scale (Wilson and Wiysonge, 2020). Marco-Franco *et al*., while discussing fake news and vaccination, called for a public health education approach at administrative and healthcare levels to fight fake news and improve protection against COVID-19 (Marco-Franco *et al*., 2021). In a recent randomized controlled trial conducted both in the USA and UK, fewer people were shown likely to be ‘definitely’ accepting a vaccine for herd immunity, and ‘scientific-sounding’ misinformation, especially when repetitive, it was shown to reduce vaccination intent (Loomba *et al*., 2021). This is of particular worry in the Asian context, where not only COVID-19 cases are surging, but the public healthcare infrastructure and limited resources in many low- and middle-income countries (LMIC) have to fight an added burden of snowballed misinformation. While arguably the right to get vaccinated or not is highly individualistic, the spread of biased opinions, deliberate disinformation, and fake news against evidence can distort public perceptions and decision making related to vaccines. This has detrimental effects on public health.

## COVID-19 and psychiatric disorders: The ‘double jeopardy’

Psychiatric illnesses and the SARS-COV-2 infection have been shown to have a mutual association, i.e., those with mental illnesses tend to be more vulnerable to COVID-19, as well as the infection *per se* may lead to psychosocial outcomes (Taquet *et al*., 2021b). A large-scale retrospective cohort analysis from the UK found out that about 12% of those tested COVID positive between March to July 2020, had a pre-existing psychiatric illness, including depression, anxiety, psychoses, and stress-related disorders (assessed both in-patient admissions and primary care visits due to psychiatric illness since 1981, using the database). There was an increased risk of COVID-19 infection (0.9% *v*. 0.4%) and related in-patient admission due to severity (0.7% *v*. 0.3%), and COVID-deaths (0.2% *v*. 0.1%) among those with pre-pandemic psychiatric condition compared to those without, with odds ratios of around 1.5 for infection and 2 for death, after adjusting for socio-demographic covariates. The researchers also found a higher risk in the older age group (>64 years). Total number of pre-existing psychiatric disorders had a significant association with current COVID-19 infection. Though the odds of infection were lower in primary care population, they were still statistically significant for the presence of psychiatric illnesses. These associations were found to be independent of body mass index, metabolic status, smoking, though compounded by the presence of physical comorbidities (Yang *et al*., 2020). Another case−control study reported a higher risk of COVID-19 diagnosis in patients with bipolar disorder, depression, schizophrenia (Wang *et al*., 2021b). Earlier studies have shown association between psychiatric conditions and symptoms such as stress, and severe infection, including respiratory problem (Pedersen *et al*., 2010; Song *et al*., 2019; Cohen, 2021). There are several hypotheses regarding the underlying mechanism, which may be persistently raised hypothalamo−pituitary−adrenal (HPA) axis activity due to maladaptive stress response (as observed in depression and anxiety) leading to altered glucocorticoid cascade, and further, heightened inflammation and reduced immunity (Glaser and Kiecolt-Glaser, 2005; Wohleb *et al*., 2016; Sadock *et al*., 2017). Excessive proinflammatory cytokines and chemokines, and lowered bodily immune response are known to worsen infection status (Cohen *et al*., 2012; Morey *et al*., 2015). In addition, medical comorbidities are common in persons with mental illness (PMI), making them more susceptible to infection. In severe mental illnesses (SMI) such as schizophrenia, depression, bipolar disorder, medical illnesses, including diabetes mellitus, cardiovascular disorders, respiratory tract infections, neurocognitive disorders, obesity and smoking, are found to be higher than in general population, even after controlling for the contribution of psychotropic medications (Miller *et al*., 2006; DE Hert *et al*., 2011; Sadock *et al*., 2017; Zolezzi *et al*., 2017). Further risk factors mediating the link between psychiatric illnesses may be socio-environmental factors like homelessness, reduced access to healthcare, lack of information, high-risk living conditions, etc. (Wang *et al*., 2021b). These factors are more important in the LMICs where poverty is severe. The nexus between poverty, homelessness, and SMIs presents a unique challenge in the LMICs as explained by theories of social drift and social causation (Lund and Cois, 2018). The mental health services are far from adequate, and often fail to cover the most vulnerable population (Narasimhan *et al*., 2019). The burden of SMIs, as a result, is not only substantial, but also difficult to manage. Various such intersections between COVID-19 and SMI are summarized in Table 1.

**Table 1.** Considerations for individuals with SMI during the COVID-19 pandemic

Increased chance of contracting the infection Worsening neuropsychiatric sequelae of the infection (delirium, cognitive decline, depression, anxiety, psychosis, adjustment disorders) Stress-vulnerability diathesis (relapse of psychiatric complaints) Possible pharmacological interactions (more with SSRIs, TCAs, stimulants, opioids, BZDs, clozapine) Reduced adherence to COVID appropriate behaviors Increased biopsychosocial frailty (due to stress, fear, social distancing) Impaired quality of life Reduced mental healthcare visits (logistic difficulties and transportation issues during lockdown) Prone to misinformation Increased stigma and discrimination Health inequality (healthcare access and services) Specific vulnerabilities of high-risk populations (elderly, sexual minorities, low SES, migrants, homeless) Dual-burden on the mental healthcare infrastructure of LMIC Reduced access and coverage of vaccination KAP gap related to vaccination Risk of abuse and institutionalization Possible long-term neuropsychiatric complications (under research)

SSRI, Selective Serotonin Reuptake Inhibitors; TCA, Tricyclic Antidepressants; BZD, Benzodiazepines; SES, Socioeconomic status; LMIC, Low-and-middle-income countries; KAP, Knowledge Attitude Practice.

Source: Taquet *et al*. (2021b), Banerjee (2020), Choi (2021), The Lancet Infectious diseases (2020), Vindegaard and Benros (2020).

The other direction of association is also significant. A study found 2.1 odds of developing a psychiatric disorder within 14−90 days of COVID-19 infection, with the highest odds of anxiety disorders, dementia, and sleep disturbances. Incidence of any psychiatric diagnosis was 18.1% within the same duration (Taquet *et al*., 2021b). This risk was again irrespective of medical comorbidities, though socioeconomic conditions could play a part. Another study found the need for health information and perceived pandemic impact as two important mediators between physical symptoms resembling COVID-19 infection and psychiatric problems, where public mental health education and interventions have a significant role (Wang *et al*., 2021a). Similar neuroimmunoendocrine pathways are said to be implicated in this relation (Raony *et al*., 2020). These findings emphasize the bidirectional relation between COVID-19 infection vulnerability and severity, and mental illnesses. In addition to this, it is important to mention here the role of digital misinformation in affecting the mental health of individuals. The ‘digital infodemic’, mentioned earlier, is responsible for multiple psychological issues such as anxiety, fear, agitation, uncertainty, noncompliance to precautions, stigma, due to the huge misinformation load. The increased screen time and unhealthy technology use, found in the population at large, further contribute to the mental health problems (Banerjee and Rao, 2020; Banerjee and Meena, 2021). Thus, it will be apt to say that preventing COVID-19 infection is instrumental in reducing incident psychiatric disorders, in improving the outcome of the infection in PMI, and fighting the battle of misinformation and its adverse consequences.

## Unique roles and opportunities

MHPs, especially psychiatrists, are uniquely positioned to deal with some of these public health challenges in the following ways:

1. **Prioritizing vaccinations in individuals with SMI and substance use disorders (SUD) in public health:** Literature mentions people with SMI and SUD to be vulnerable groups besides their propensity for physical comorbidity, and recommends urgent vaccination in them (Mazereel *et al*., 2021). As we have discussed in the previous section, studies have shown bidirectional associations between pre-existing psychiatric disorder and severity of SARS-CoV-2 infection as well as illness-related hospitalization, morbidity and mortality (Yang *et al*., 2020; Taquet *et al*., 2021b; Wang *et al*., 2021b). Dysregulated immunity, chronic stress and sleep disturbances are a few other additional vulnerabilities for SMI-COVID-19 interactions (Mazereel *et al*., 2021). Social factors such as poverty, homelessness, stigmatization, discrimination, and social stereotypes not only contribute to the COVID-19-related stress, but also to the access to healthcare and vaccination (Torales *et al*., 2020). The older people deserve special mention here, who are susceptible both to the biological as well as psychosocial offshoots of COVID-19. They often tend to get ‘marginalized’ with reduced healthcare access and are at an enhanced risk of mortality and morbidity due to the pandemic (Banerjee *et al*., 2020). This is compounded by mental illness, sensory and cognitive deficits. Prioritizing vaccination for them is imperative. Psychiatrists have multifold roles in COVID-19 vaccination for PMI, including calling for prioritization of vaccination in SMI, assessing and managing socio-environmental mediators, educating the general populations, PMI, caregivers, and other stakeholders regarding the need for urgent vaccination, and lending their expertise regarding the effect of SMI on COVID-19 vaccination as well as interactions with psychotropics.
2. **Addressing the barriers and advocating the enablers for vaccination access in people with SMI:** Barriers to vaccination include attitudinal (e.g. beliefs about vaccines, trust in healthcare system, motivation), socio-environmental (e.g. stigma, cultural unacceptability, misinformation-led cognitive bias), and structural (access to healthcare including transportation, cost, etc.) barriers (Fisk, 2021; Who Technical Advisory Group on Behavioural and Insights and Sciences for Health, 2020). MHPs can liaise with other specialties in improving vaccine awareness and education, creating resources for vaccination programs, promoting engagements, monitoring of vaccination drives, and discussing with administrators for policies related to cost and access to vaccines in people with SMI. The ideal center for vaccination can also be decided: inpatient or residential facilities, community mental health centers, primary care clinics, public health clinics, at home or local pharmacies. MHPs can participate in vaccine awareness, education, and destigmatization in the general population as well.
3. **Improving public and preventive psychiatry:** COVID-19 has already been mentioned as an opportunity to revamp the mental healthcare services in many nations. MHPs can play the role of ‘integrators’ of vaccination drive in the community, as it bolsters awareness, sense of security, and psychological safety in individuals (Dandona and Sagar, 2021). This will help integrate mental healthcare for the ongoing pandemic crisis as a public health priority together with the vaccination programs.
4. **Fighting fake news and misinformation:** As already mentioned, MHPs can act in a unique capacity in this regard. Various psychological processes like cognitive biases, social modelling, misattribution, fear-reasoning, etc. play in the genesis of fake news (Banerjee and Rao, 2020). Similar misinformation and stigma exist in relation to mental illness as well. It has been shown by Zhang *et al*. that fact-checking information by health professionals, especially through social media, can have beneficial effects on public attitudes towards vaccination (Zhang *et al*., 2021). Public education and awareness programs can help modify and address some of the public misconceptions similar to stigma-mitigating strategies and psychoeducation used in mental health practice. This will help curb the mistrust, paranoia and uncertainty that arise out of this misinformation. Further research is also warranted into the psychology of misinformation and approaches to curb them.
5. **Managing the ‘worried well’:** The concept of ‘worried well’ encompasses those unexposed but developing some symptoms, those seeking medical advice due to fear of exposure, and those experiencing anxiety after any sort of trauma. This category needs a separate mention because of the significant associated psychological distress, as well as the high usage of healthcare services. With or without any diagnosable psychiatric syndromes such as somatoform disorder, hypochondriasis, these patients experience sufficient stress and anxiety, worsening their mental health and general well-being (Chatterjee *et al*., 2020). This presentation may be relevant to the vaccination also, including worries about vaccine and its side effects, perceived severity and lethality of post-vaccine physical symptoms, fear of vaccine-related complications and refusal of vaccine, thereby increasing the public health risk. Thus, adequate management, often in form of psychoeducation and supportive treatment, is necessary to reduce the burden on both the medical care, patient, and public health consequences (KINI *et al*., 2020).
6. **Empowering the vulnerable groups:** Age and gender minorities, frontline workers, poor socio-economic class, migrants, homeless, and individuals with SMI all are high-risk populations for COVID-19. As many of them have associated psychosocial morbidity as well, MHPs can serve as advocators for their mental as well as physical wellbeing, helping them access an early vaccination drive. They can also assist public health officials in estimating decisional capacity and consent for vaccination in these individuals, so that it stays respectful to their autonomy.
7. **Role modeling:** The American Psychiatric Association (APA) has issued a statement calling on the Department of Health and Human services to issue guidance advising states to include mental health and addiction service providers to be included in priority groups for vaccinations (American Psychiatric Association, 2021). Psychiatrists frequently serve as a prolonged and trusted point of contact between people with mental illness and the health system. Thus, they can serve as role models to alleviate the doubts and improve faith regarding vaccination in the PMIs. Though it is always a personal choice whether to get vaccinated, but MHPs can always help take an informed decision, both for themselves and the community.
8. **Caveats in low- and-middle-income countries (LMIC):** The infection burden is found to be higher in the LMIC along with mortality (Choi, 2021). COVID-19 has compounded the pre-existing difficulties in these countries, such as poor sanitation, crowded living conditions, endemic infections, and a substantial inadequacy of healthcare. Strict public health measures of lockdown, curfew may impact the socio-economically disadvantaged people significantly, thus, adding to the challenges. The vaccine supply and distribution are also differential across high-income countries and LMICs (Choi, 2021). Along with the shortage and inequitable distribution of vaccines, specific caveats encountered in LMICs, as mentioned before, are low awareness and hesitancy regarding vaccination, misinformation, stigma, lack of adequate number of professionals for the population, to name a few.

MHPs, therefore, here have a more elaborate part to play. There was an article in 2019 pointing out the psychiatrist−patient ratio in India. It has been found from several registers that the ratio is around 0.75 per 100000 population in India, whereas the desirable ratio is at least three psychiatrists per 100000 population. They estimated that 2700 more psychiatrists are required in the next 10 years to bridge this gap (Garg *et al*., 2019). In view of around 700 psychiatrists post-graduating every year, the number is difficult to reach in the next couple of years. In this pandemic, this dearth can be managed by training primary care physicians and other professionals in mental health. A significant number of patients present to the primary care and to other disciplines (gastroenterology, neurology, endocrinology, dermatology, general medicine, etc.) with various psychological problems. Thus, training and educating the professionals and the grassroot workers like Anganwadi workers, multipurpose health workers in the basic assessment and management of psychiatric issues, identification of appropriate referral requirements, and in vaccination advocacy along with addressing vaccine-related barriers will be important in supporting and promoting mental health in relation to the pandemic as well as vaccination. The telemedicine guidelines released by the Government of India in 2020 can be used in this context by psychiatrists and other MHPs alike, to overcome the geographical barrier to the access to mental health education, awareness, training, and treatment (The Ministry of Health and Family Welfare, Government of India, 2020).

## Conclusion

The pandemic is far from being over and so is our role as psychiatrists and other MHPs. The rising burden of psychosocial morbidity and need to understand the neuropsychiatric underpinnings of COVID-19 have re-emphasized the vital role of the MHPs. The mutual association between the incidence, severity, and mortality of the infection, and psychiatric morbidity is likely to affect the public health at large. In addition to prioritizing vaccination needs of the mentally ill individuals, MHPs can be instrumental in addressing socio-environmental barriers and needs of other high-risk communities, like migrants, homeless, frontline workers. It is imperative that this is just the tip of iceberg. As the pandemic continues, vulnerable groups of population like the elderly PMI, homeless PMI, religious and sexual minority, etc. will continue to face the difficulties, which when accumulated, may lead to more deleterious mental health consequences. Hence, the role of MHPs, including in treating psychiatric comorbidities and advocating vaccination goes a long way. In the role pertaining to the vaccination, MHPs can serve as ‘vaccine ambassadors’ to help community outreach, promote equitable vaccination distribution, battle medical misinformation, and use this opportunity to integrate psychosocial and public health priorities for those living with mental illness. In order to target the health inequity and discrimination faced by people with SMI coupled with their additional risks, the authors urge the global mental health fraternity to tailor these crucial roles with respect to COVID-19 vaccination based on the regional needs and contexts.
